# Adjuvant Chinese Herbal Products for Preventing Ischemic Stroke in Patients with Atrial Fibrillation

**DOI:** 10.1371/journal.pone.0159333

**Published:** 2016-07-18

**Authors:** Yu-Chiang Hung, Yu-Chen Cheng, Chih-Hsin Muo, Hsienhsueh Elley Chiu, Chun-Ting Liu, Wen-Long Hu

**Affiliations:** 1 Department of Chinese Medicine, Kaohsiung Chang Gung Memorial Hospital and Chang Gung University College of Medicine, Kaohsiung, Taiwan; 2 School of Chinese Medicine for Post Baccalaureate, I-Shou University, Kaohsiung, Taiwan; 3 Management Office for Health Data, China Medical University Hospital, Taichung, Taiwan; 4 Kaohsiung Medical University College of Medicine, Kaohsiung, Taiwan; 5 Fooyin University College of Nursing, Kaohsiung, Taiwan; Women’s Hospital, School of Medicine, Zhejiang University, China. 310006, CHINA

## Abstract

**Objective:**

Chinese herbal products (CHPs) are widely used for atrial fibrillation (AF) in Taiwan. We investigated the effect of adjuvant CHPs in preventing ischemic stroke in patients with AF.

**Methods:**

Taiwanese patients in the Health Insurance Database newly diagnosed with AF during 2000–2011 were enrolled. Medication treatment with/without CHPs was administered within 7 days after the AF diagnosis. The clinical endpoint was an ischemic stroke. The Chi-square test, Fisher’s exact test, and Student *t* test were used to examine differences between the traditional Chinese medicine (TCM) and non-TCM cohorts. Cox proportional hazard regression was used to assess the risk for ischemic stroke between two cohorts.

**Results:**

Three hundred and eleven patients underwent TCM treatment and 1715 patients did not. Compared to non-TCM users, TCM users had a lower incidence of stroke (12.59% vs. 1.93%, respectively) and lower risk of stroke [CHA_2_DS_2_-VASc score = 0–2 (hazard ratio = 0.20; 95% confidence interval = 0.06–0.65)]. Compared to non-TCM users, the stroke risk was significantly lower in TCM users with AF who were female or younger than 65 years, but not in males, people more than 65 years old, or people with comorbidities. Compared to TCM users, non-TCM users who received conventional treatment had a higher ischemic stroke risk. The risk for AF-related hospitalization was significantly lower in TCM users (0.64%) than in non-TCM users (38.1%).

**Conclusions:**

Users of TCM with AF have a lower risk of new-onset ischemic stroke. Therefore, adjuvant CHP therapy may have a protective effect and may be used in AF patients to prevent ischemic stroke.

## Introduction

Atrial fibrillation (AF) is characterized by disorganized, rapid, and irregular atrial activation with loss of atrial contraction and an irregular ventricular rate that is determined by AV nodal conduction [1]. It is the most common arrhythmia and significantly increases stroke risk in adults. Some risk factors for stroke are hypertension, diabetes, and hyperlipidemia [2]. The incidence of AF increases with age, so more than 5% of adults older than 70 years will experience an AF [3]. In the Western world, AF affects approximately 1%–2% of the general population [4,5]. In the Chinese population, approximately 1.4% of men and 0.7% of women have an AF [6]. Common AF-associated complications are stroke, thromboembolism, and cardiomyopathy. Cardioembolism is responsible for approximately 20% of all ischemic strokes, and the most significant cause of cardioembolic stroke is nonrheumatic (i.e., nonvalvular) AF. Patients with AF have an average annual stroke risk of approximately 5%. The risk of stroke can be estimated by calculating the CHA_2_D_2_-VASc score, [7] also called the Birmingham 2009 scheme, which is obtained by assigning one point for each of the following factors: congestive heart failure, hypertension, age older than 75 years, diabetes mellitus, female, and vascular disease, while assigning two points for a history of stroke or transient ischemic attack. A CHA_2_DS_2_-VASc score of 2 or greater indicates a high risk. The relative risk of stroke for patients with AF is 1.8- to 2.9-fold [8]. Anticoagulation therapy is conventionally used to mitigate a possible stroke risk in patients with AF [9]. However, patients with AF under anticoagulation therapy might show a sudden onset of hemorrhagic stroke or an adverse effect such as gastrointestinal or urinary tract bleeding [10].

Traditional Chinese medicine (TCM) has been widely used in the Eastern world for thousands of years. Since 1995, Chinese herbal products (CHPs) have been listed under the National Health Insurance (NHI) program in Taiwan. Individual herbs such as Dan Shen (*Salvia miltiorrhiza* Bunge, rhizome) or herbal formulas such as *zhi-gan-cao-tang* (ZGCT; a multiple composition) are used to treat heart disease [11,12]. Whether adjuvant CHP therapy effectively prevents ischemic stroke in AF patients is still unclear. We investigated the effect of adjuvant CHP therapy on preventing ischemic strokes in patients with AF. We used a nationwide cohort containing nearly 100% Taiwanese residents who received health insurance from 2000 to 2011 in Taiwan.

## Methods

### Data resources

Since March 1995, Taiwan has implemented the NHI program. Until the end of 2014, approximately 99% of the total Taiwanese population had enrolled in the NHI program (NHI research database [NHIRD]; http://nhird.nhri.org.tw/en/). The Bureau of NHI (BNHI) is contracted with 97% of hospitals and clinics throughout the nation [13]. The database provides a representative sample of data, and includes some medical information for several years and keeps track of the people whose details are entered into the sample files.

Longitudinal Health Insurance Database 2000 (LHID2000) includes information for 1 million people. The identification of insurants in the LHID2000 was recoded before being sent to researchers because of the Personal Information Protection Act. This study was approved by the Institutional Review Board of China Medical University in Central Taiwan (CMU-REC-101-012).

### Study population

**Fig 1:**
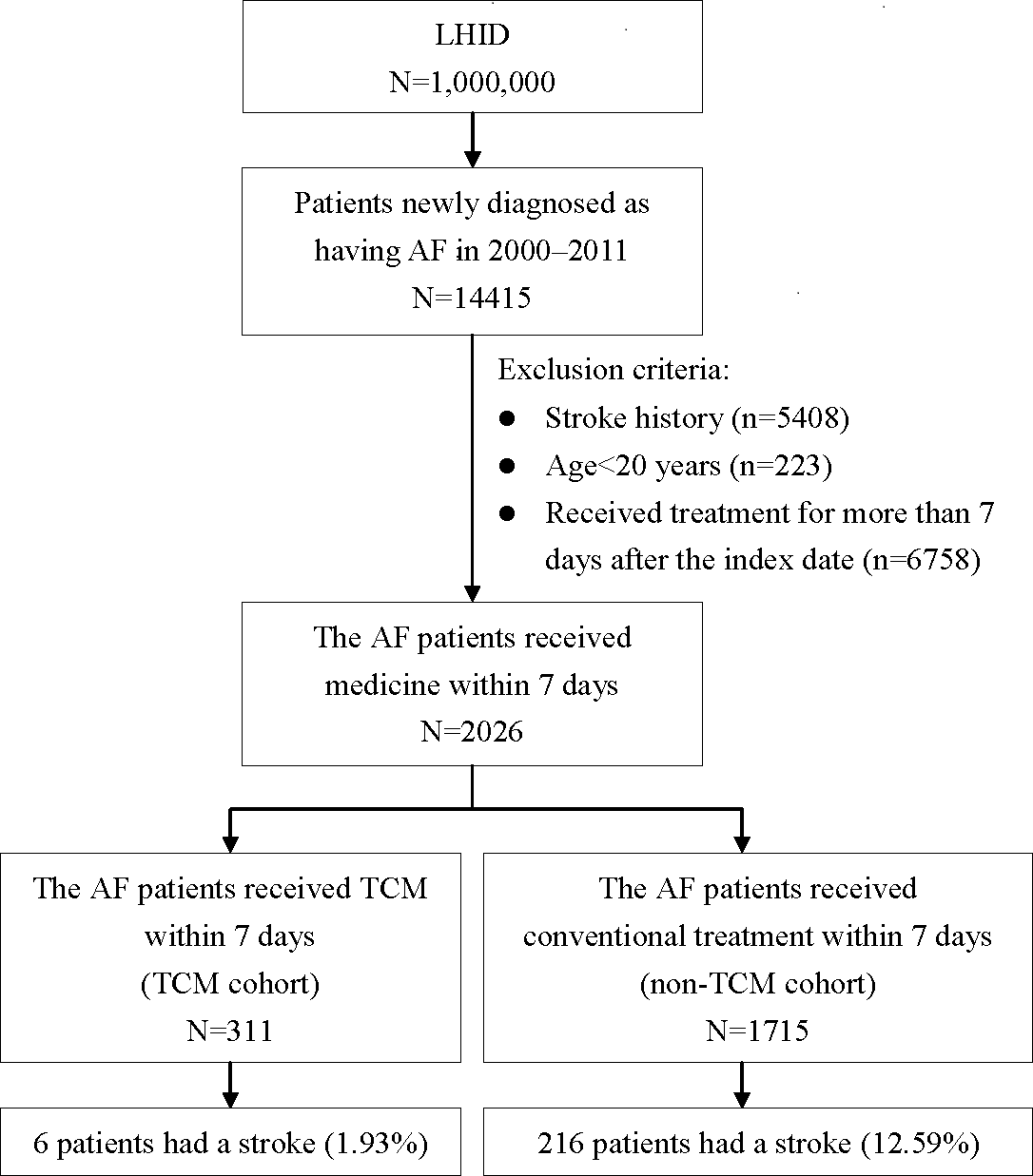
Flow chart of the study participants. AF, atrial fibrillation; LHID, Longitudinal Health Insurance Database; TCM, traditional Chinese medicine.

We collected the data for 14,415 patients with newly diagnosed AF (ICD-9-CM codes 427.31 and 427.89) from the LHID during 2000–2011. Patients with AF were excluded if they had a history of stroke (based on the *International Classification of Diseases*, *Ninth Revision*, *Clinical Modification* [ICD-9-CM] codes 430–438), were younger than 20 years, or had undergone medication treatment more than 7 days after the date of the AF diagnosis. Medication treatment was administered with/without CHPs within 7 days after the date of the AF diagnosis. AF patients were grouped into the TCM cohort (i.e., CHPs and conventional treatment) or the non-TCM cohort (i.e., conventional treatment only). The endpoint was an ischemic stroke (ICD-9-CM codes 433–438) or the end of 2011. (Fig 1)

Potential risk factors included hypertension (ICD-9-CM codes 401–405), diabetes (ICD-9-CM code 250), hyperlipidemia (ICD-9-CM code 272), congestive heart failure (CHF; ICD-9-CM codes 428, 402.01, 402.11, 402.91, 404.01, 404.11, and 404.91), and ischemic heart disease (ICD-9-CM codes 410–414, 443.89, 443.9 and 444). Complications of bleeding included hemorrhagic stroke (ICD-9-CM codes 430–432) and gastrointestinal bleeding (ICD-9-CM code 578). All risk factors were defined before the date of the AF diagnosis.

### Statistical analysis

All analyses used SAS software, version 9.3 (SAS Institute, Cary, NC). The statistical significance level was *P*<0.05 (two-tailed). The Chi-square test was used to test differences between the TCM and non-TCM cohorts, based on sex, age (i.e., younger than 65 years or 65 years and older), comorbidities, and one complication (e.g., gastrointestinal bleeding). Fisher’s exact test was used to test the differences between the two cohorts for another complication (i.e., hemorrhagic stroke). The Student *t* test was used to test the difference between the two cohorts in the mean age and CHA_2_DS_2_-VASc score. Person-years were counted in each cohort from the index date to the date of ischemic stroke (ICD-9-CM codes 433–437). Patients who did not have a stroke were followed until the date they withdrew from the program or the end of 2011, whichever was first. For each cohort, the ischemic stroke incidence was calculated by the number of ischemic strokes divided by person-years. The number of person-years was calculated by summing the number of years from the index date to the date of endpoint. Cox proportional hazard regression was used to assess the risk for ischemic stroke in the TCM cohort in comparison to the non-TCM cohort. Model 1 was adjusted for continuous age, sex, and all comorbidities. Model 2 was adjusted for the CHA_2_DS_2_-VASc score. Risks specific for age, sex, comorbidities, and CHA_2_DS_2_-VASc score were estimated. The association between ischemic stroke and different medication treatments was assessed. We also estimated the risk of emergency room (ER) visits and hospitalization due to AF within 1 year after the index date in TCM users, compared to non-TCM users in logistical regression. In the sensitivity test, we selected two subgroups of non-TCM users from the original non-TCM cohort. The first non-TCM subgroup was matched by age, sex, and comorbidity according to propensity score. The second non-TCM subgroup was matched by the propensity score, which was calculated by logistical regression, based on the CHA_2_DS_2_-VASc score (S1–S4 Tables).

## Results

We enrolled 2026 AF patients: 311 patients with CHP treatment and 1715 patients without CHP treatment. Patients in the TCM cohort, compared to the non-TCM cohort, were more often women (75.9% [n = 236] vs. 44.5% [n = 763], respectively), younger (49.4±14.3 years vs. 65.0±14 years), and had fewer comorbidities and complications such as hypertension (27.7% vs. 66.8%), diabetes (6.43% vs. 18.0%), ischemic heart disease (19.0% vs. 53.2%), CHF (1.93% vs. 24.1%), hemorrhagic stroke (0.32% vs. 2.68%), and gastrointestinal bleeding (4.5% vs. 12.8%) (Table 1). There was no difference between the two cohorts in the prevalence of hyperlipidemia. The mean CHA2DS2-VASc score was lower in the TCM users (1.49±1.11) than in the non-TCM users (2.88±1.68).

**Table 1:** The Comorbidities, CHA2DS2-Vasc Scores, and Complications between the TCM and Non-TCM Groups.

	TCM (N = 311)		Non-TCM (N = 1715)		*P* value
	n	%	n	%	
Comorbidities					
Hypertension	86	27.7	1145	66.8	<0.0001
Diabetes	20	6.43	309	18.0	<0.0001
Hyperlipidemia	86	27.7	567	33.1	0.06
Ischemic heart disease	60	19.0	913	53.2	<0.0001
CHF	6	1.93	414	24.1	<0.0001
Mean CHA2DS2-VASc score (SD)	1.49	(1.11)	2.88	(1.68)	<0.0001
Complications					
Hemorrhagic stroke	1	0.32	46	2.68	0.007^1^
Gastrointestinal bleeding	14	4.50	220	12.8	<0.0001^2^

CHA2DS2-VASc, congestive heart failure, hypertension, age ≥75, diabetes mellitus, prior stroke or transient ischemic attack–vascular disease, age 65 to 74, female; CHF, congestive heart failure; SD, standard deviation; TCM, traditional Chinese medicine.

The CHA2DS2-VASc score excludes a previous stroke.

^1^ Based on Fisher’s exact test.

^2^ Based on the Chi-square test.

During the mean 5.17 follow-up-year period, the incidence of ischemic stroke was lower in the TCM cohort (5.25/1000 person-years) than in the non-TCM cohort (23.14/1000 person-years) (Table 2). After adjusting for age, sex, and comorbidities, the hazard ratio for ischemic stroke was significant at 0.39 (95% CI = 0.17–0.90). The other adjusted CHA2DS2-VASc score for ischemic stroke was 0.33 (95% CI = 0.15–0.75). The TCM users had a significantly lower risk of stroke, compared to non-TCM users who were women [HR = 0.20 (95% CI = 0.17–0.90) in model 1; HR = 0.19 (95% CI = 0.05–0.83) in model 2] or younger than 65 years [HR = 0.10 (95% CI = 0.01–0.07) in model 1; HR = 0.09 (95% CI = 0.01–0.63) in model 2]. In addition, TCM users had a significantly lower ischemic stroke risk [HR = 0.20 (95%CI = 0.06–0.65)] than non-users in patients with a CHA_2_DS_2_-VASc score of 0–2. There were no significant differences between patients who were male, older than 65 years, had comorbidities, and a CHA2DS2-VASc score > 2. In the sensitivity analyses, TCM users and non-TCM users had the same trends, regardless of which matched criteria were used.

**Table 2:** The Incidence and Hazard Ratios for Ischemic Stroke in the TCM Cohort, Compared to the Non-TCM Cohort.

	TCM			Non-TCM			TCM vs. non-TCM HR (95% CI)	
	Event (no.)	PY	Rate^#^	Event (no.)	PY	Rate^#^	Model 1	Model 2
Overall	6	1143	5.25	216	9332	23.14	0.39 (0.17–0.90)*	0.33 (0.15–0.75)**
Sex								
Women	2	825	2.43	113	4261	26.52	0.20 (0.05–0.83)*	0.19 (0.05–0.78)*
Men	4	318	12.58	103	5071	20.31	0.86 (0.31–2.36)	0.77 (0.28–2.13)
Age (years)								
<65	1	966	1.04	62	4846	12.79	0.10 (0.01–0.70)*	0.09 (0.01–0.63)*
65+	5	177	28.27	154	4486	34.33	0.91 (0.37–2.25)	0.96 (0.39–2.36)
Comorbidity								
No	2	592	3.38	21	1634	12.86	0.48 (0.10–2.24)	
Yes	4	551	7.27	195	7699	25.33	0.38 (0.14–1.03)	
CHA2DS2-VASc score ^†^								
< 2	3	953	3.15	64	4440	14.41		0.20 (0.06–0.65)**
≥ 2	3	190	15.80	152	4893	31.07		0.51 (0.16–1.59)

CHA2DS2-VASc, congestive heart failure, hypertension, age ≥75, diabetes mellitus, prior stroke or transient ischemic attack–vascular disease, age 65 to 74, female; HR, hazard ratio; PY, person-year; TCM, traditional Chinese medicine.

Model 1, adjusted for age, sex and comorbidities.

Model 2, adjusted for the CHA2DS2-VASc score.

* *P*<0.05

** *P*<0.01

^†^ The data are based on a univariate model.

^#^ per 1000 person-years.

The proportion of AF-related ER visits within 1 year after the AF diagnosis was lower in TCM users than in non-users (14.5% vs. 15.3%, respectively; odds ratio, 0.94 in model 1 and 0.87 in model 2). The risk for AF-related hospitalization within 1 year of diagnosis was also significantly lower in TCM users (0.64%) than in non-users (38.1%) (odds ratio, 0.01 in model 1 and 0.02 in model 2) (Table 3). The top three combined diseases responsible for AF-related hospitalization were heart failure (10.1%), ischemic heart disease (10.1%), and diabetes (5.5%). The top three combined diseases responsible for AF-related ER visits were heart failure (13.7%), ischemic heart disease (12.7%), and symptoms involving the cardiovascular system (5.86%). The annual total medical cost was 1988 NTD (New Taiwan Dollar) and 61310 NTD in TCM and non-TCM cohort during the study period. The most common formula and single CHPs used for AF treatment in Taiwan were ZGCT (5.42%; Table 4) and Dan Shen (3.95%; Table 4).

**Table 3:** The Odds Ratio for Emergency Room Visits and Hospitalization due to Atrial Fibrillation within 1 Year after the Index Date in the TCM Cohort, Compared to the Non-TCM Cohort.

	TCM		Non-TCM		TCM vs. non-TCM OR (95% CI)		
	n	%	n	%	Crude	Model 1	Model 2
ER	45	14.5	262	15.3	0.94 (0.67–1.32)	0.87 (0.59–1.28)	0.86 (0.60–1.23)
Hospitalization	2	0.64	653	38.1	0.01 (0.003–0.04)***	0.02 (0.004–0.07)***	0.01 (0.003–0.06)***

CHA2DS2-VASc, congestive heart failure, hypertension, age ≥75, diabetes mellitus, prior stroke or transient ischemic attack–vascular disease, age 65 to 74, female; ER, emergency room; OR, odds ratio; TCM, traditional Chinese medicine.

Model 1, adjusted for age, sex, and comorbidities.

Model 2, adjusted for CHA2DS2-VASc score.

*** *P*<0.0001.

**Table 4:** The top 5 single and formula CHPs prescribed by TCM physicians for treating patients with AF from 2000 to 2010 in Taiwan.

Single CHP	n	%	Formula CHP	n	%
Dan Shen (*Salvia miltiorrhiza* Bunge, rhizome)	433	3.95	Zhi-gan-cao-tang	594	5.42
Tian Hua Fen (*Trichosanthes kirilowii* Maxim., rhizome)	290	2.65	Tian-wang-bu-xin-dan	509	4.64
Yuan Zhi (*Polygala tenuifolia* Willd., root)	235	2.14	Fu Fang Dan Shen Pian	205	1.87
Da Huang (*Rheum palmatum* L., rhizome)	233	2.13	Qing-xin-lian-zi-yin	162	1.48
Sha Ren (*Amomum villosum* Lour., fruit)	226	2.06	Xue-fu-zhu-yu-tang	155	1.41

CHP, Chinese herbal product; TCM, traditional Chinese medicine; AF, atrial fibrillation.

## Discussion

In this nationwide cohort analysis, we showed that adjuvant CHP therapy could reduce the incidence of new ischemic stroke in AF patients who are female, younger than 65 years, and have a CHA_2_DS_2_-VASc score less than 2 (i.e. low risk for ischemic stroke). A previous study showed that female patients with AF and a CHA_2_DS_2_-VASc score of 1 had a higher risk for ischemic events, compared to non-AF patients [14]. Some researchers have found a lower incidence of stroke in pre- menopausal women, but a higher incidence of stroke in post-menopausal women compared with men. This phenomenon may be due to the potential neuroprotective benefits of hormones, most notably estrogen [15,16]. However, estrogen had no dominant effects in female patients with AF. They have been noted to have greater thromboembolic risk than male patients with AF [17,18], which may result from biological factors, including increased hypertension, renal dysfunction, and hyperthyroidism, in female patients with AF. Our results showed that patients with AF in the TCM cohort, compared to the non-TCM cohort, were more often women (75.9% [n = 236] vs. 44.5% [n = 763]). Female patients with AF in the TCM group having lower risk of stroke than male patients with AF may be due to a stronger response to integrated TCM and conventional Western medicine. (TCM group were treated with TCM and conventional Western medicine; non-TCM group were only treated with conventional Western medicine). The other result also showed there were more younger patients with AF in the TCM cohort than in the non-TCM cohort (49.4±14.3 years vs. 65.0±14 years). A previous study found age-related changes in brain support cells [19]. Younger patients with AF may have fewer comorbidities and a stronger response to integrated TCM and conventional Western medicine. These results could also support the benefit of adjuvant CHP therapy in our study. We also found in the non-TCM cohort that the greater the number of medications, the higher was the incidence of stroke. Multiple medications to treat AF may indicate a greater severity of AF.

A previous cohort study revealed that approximately one in eight users of western medicine (e.g., antiplatelets, anticoagulants, or digoxin) was concomitantly prescribed CHPs in the Taiwanese population [20]. In our study, the most used single CHP and formula CHP in AF patients were Dan Shen (*Salvia miltiorrhiza* Bunge, rhizome) and ZGCT, respectively. Dan Shen is a very versatile Chinese herbal drug that has been used for hundreds of years to treat ailments such as cardiovascular diseases [21,22]. Pharmacological studies indicate that Dan Shen acts against atherosclerotic vascular disease by inhibiting smooth muscle proliferation [23,24], and is used for dilating the cardiocerebral vessels, suppressing the aggregation of platelets, activating circulation, dispersing blood stasis, protecting from ischemic reperfusion injury, and enhancing the tolerance of ischemic tissue to hypoxia [25,26]. In a recent Chinese study, Dan Shen reduced the incidence of arrhythmias from 30% to 18% [27]. Dan Shen has also been used as a standard treatment clinically for cerebrovascular diseases, which include stroke. The neuroprotective mechanisms of Dan Shen through antiapoptotic, anti-oxidant, anti-inflammatory effects were predicted by several studies [28]. However, some systematic reviews of randomized controlled trials failed to support its efficacy in improving disability after ischemic stroke [29,30]. Nevertheless, these results may be because of the unclear methodological quality of these identified trials [31].

For thousands of years in the Chinese community, ZGCT has been a representative formula to treat arrhythmia in patients with a knotted irregular pulse and severe palpitations [32]. Several clinical trials show that ZGCT may improve heart palpitations, shortness of breath, insomnia, and fatigue in patients with premature ventricular contractions [33]. In Taiwan, ZGCT is widely used alone or in combination with antiarrhythmic drugs as an alternative, effective treatment for AF. The possible mechanisms of frequently used CHPs (single and formula) for AF are presented in Table 5.

**Table 5:** Possible mechanisms of frequently used CHPs for AF from 2000 to 2010 in Taiwan.

	Known active herb constituents and formula ingredients	Possible pharmacological effects on AF
Formula CHPs		
Zhi-gan-cao-tang	(Zhi Gan Cao) *Glycyrrhiza uralensis* Fisch^†^., root and rhizome, honeyed; (Ren Shen) *Panax ginseng* C.A.Mey., root; (Gui Zhi) *Cinnamomum cassia* (L.) J.Presl, twig; (Sheng Jiang) *Zingiber officinale* Roscoe, fresh rhizome (E Jiao) *Equus asinus* L., skin; (Sheng Di Huang) *Rehmannia glutinosa* (Gaertn.) DC., root; (Mai Dong) *Ophiopogon japonicus* (Thunb.) Ker Gawl., rhizome; (Huo Ma Ren) *Cannabis sativa* L., seed; (Da Zao) *Ziziphus jujuba* Mill., fruit	Benefits *Qi* and nourishes *Yin*, tonifies *Yang*, nourishes blood and reduces palpitations. Blocks different ion channels to shorten the APD (action potential duration) of ventricular muscle cells and increases self-discipline[11]; Improves heart *Qi* deficiency[34], warms heart *Yang*, improves blood circulation, and removes blood stasis[35]
Tian-wang-bu-xin-dan	(Tian Men Dong) *Asparagus cochinchinensis* (Lour.) Merr., root; (Ren Shen) *Panax ginseng* C.A.Mey., root; (Fu Ling) *Poria cocos* (Schw.) Wolf, sclerotia; (Xuan Shen) *Scrophularia ningpoensis* Hemsl., root; (Dan Shen) *Salvia miltiorrhiza* Bunge, rhizome; (Yuan Zhi) *Polygala tenuifolia* Willd., root; (Jie Geng) *Platycodon grandiflorus* (Jacq.) A.DC., root; (Dang Gui) *Angelica sinensis* (Oliv.) Diels, root; (Wu Wei Zi) *Schisandra chinensis* (Turcz.)Baill., fruit; (Mai Dong) *Ophiopogon japonicus* (Thunb.) Ker Gawl., rhizome; (Bai Zi Ren) Platycladus orientalis (L.) Franco, seed; (Suan Zao Ren) Ziziphus jujuba Mill., seed; (Sheng Di Huang)*Rehmannia glutinosa* (Gaertn.) DC., root	Nourishes *Yin* and blood, and calms the heart to tranquilize the mind[36]; Prevents premature ventricular contractions[37] and improves cardiovascular neurosis[38]; Reduces the mortality rate associated with myocardial infarction, improves palpitations and irregular heartbeat[39]
Fu Fang Dan Shen Pian	(Dan Shen) *Salvia miltiorrhiza* Bunge, rhizome; (San Qi) *Panax notoginseng* (Burkill) F.H.Chen, root; (Bing pian) Borneolum Syntheticum	Reduces chest tightness, palpitation and angina due to heart blood stasis[40]; Protects cardiomyocytes against myocardial ischemia/reperfusion injury and inhibits apoptosis by activating the Akt-eNOS signaling pathway[41]
Qing-xin-lian-zi-yin	(Huang Qin) *Scutellaria baicalensis* Georgi, root; (Mai Dong) *Ophiopogon japonicus* (Thunb.) Ker Gawl., rhizome; (Di Gu Pi) *Lycium chinense* Mill., root bark; (Che Qian Zi) *Plantago asiatica* L., seed; (Chai Hu) *Bupleurum chinense* DC., root; (Gan Cao) Glycyrrhiza uralensis Fisch., root; (Lian Zi) *Nelumbo nucifera* Gaertn., seed; (Fu Ling) *Poria cocos* (Schw.) Wolf, sclerotia; (Huang Qi) *Astragalus propinquus* Schischkin, root; (Dang Shen) *Codonopsis pilosula* (Franch.) Nannf., root	Treats insomnia and kidney disease caused by heart fire and both deficiencies of *Qi* and *Yin*[35,42]
Xue-fu-zhu-yu-tang	(Dang Gui) Angelica sinensis (Oliv.) Diels, root; (Sheng Di Huang) *Rehmannia glutinosa* (Gaertn.) DC., root; (Tao Ren) *Prunus persica* (L.) Batsch, seed; (Hong Hua) *Carthamus tinctorius* L., flower; (Zhi Ke) *Citrus × aurantium* L., ripe fruit; (Chi Shao Yao) *Paeonia lactiflora* Pall., root; (Chai Hu) *Bupleurum chinense* DC., root; (Gan Cao) Glycyrrhiza uralensis Fisch., root; (Jie Geng) *Platycodon grandiflorus* (Jacq.) A.DC., root; (Chuan Xionɡ) *Ligusticum striatum* DC., rhizome; (Niu Xi) *Cyathula officinalis* K.C.Kuan, root	Alleviates coronary artery diseases[43], thromboembolic stroke, atherosclerosis and hyperlipidemia[44] caused by blood and *Qi* stasis; Shows neuroprotective effects by inhibiting HIF-1 α and TNF-α, followed by the inhibition of inflammatory responses (i.e., iNOS) and apoptosis (active caspase-3)[45]
Single CHPs		
Dan Shen	*Salvia miltiorrhiza* Bunge, rhizome	Shows anti-atherosclerotic, anti-cardiac hypertrophic, anti-oxidant, and anti-arrhythmic effects by promoting blood circulation and provides relief from blood stasis[46]; Improves microcirculation, causes coronary vasodilatation, suppresses the formation of thromboxane, inhibits platelet adhesion and aggregation, and protects against myocardial ischemia[21]
Tian Hua Fen	*Trichosanthes kirilowii* Maxim., rhizome	Treats hypertension, hyperlipidemia,[47] and blood plasma viscosity caused by blood stasis[48]
Yuan Zhi	*Polygala tenuifolia* Willd., root	Shows sedative, antipsychotic, expectorant, and anti-inflammatory effects and anti-thrombotic activity[49–51]
Da Huang	*Rheum palmatum* L., rhizome	Shows Anti-inflammatory and anti-oxidative activity[52,53]; Decreases the incidence of brain injury after ischemic stroke[54]; Removes accumulation with purgation, clear heat and purge fire, cools the blood and removes toxins, expels stasis to unblock the meridian and drain dampness[55]
Sha Ren	*Amomum villosum* Lour., fruit	Alleviates spleen deficiency and Qi stagnation [56,57]; Shows anti-oxidative and anti-inflammatory activity [58,59]

CHP, Chinese herbal product; AF, atrial fibrillation; TCM, traditional Chinese medicine.

^†^All of botanical plant names were based on “The Plant List (http://www.theplantlist.org/)”.

Dan Shen may interact with the anticoagulant action of warfarin. Patients receiving warfarin therapy may show gross overanticoagulation and bleeding complications when taking Dan Shen [60]. Dan Shen may be able to bind with warfarin to affect its absorption [61]. Some review articles have listed many CHPs that interact with warfarin by influencing absorption, metabolizing enzymes, protein binding, platelet function, vitamin K cycle, or coagulation cascade [62,63]. Thus, it is necessary to have an interval of 1 hour between taking CHPs and taking a Western medication. It is important to monitor the prothrombin time or the international normalized ratio in these patients.

After ischemic stroke or transient ischemic attack, a patient should not have oral anticoagulants combined with antiplatelet therapy. The most recent advice by the American Heart Association for treating stroke in AF patients proposes this. However, this treatment is reasonable in patients with clinically apparent coronary artery disease, particularly patients with acute coronary syndrome or stent placement [64]. We excluded patients with a previous stroke history in this cohort; however, the prevention of new onset ischemic stroke on AF patients using CHPs was significant. Our results suggested that TCM reduces the severity of AF, which subsequently reduces the risk of stroke and were consistent with previous reports in which adjuvant CHP medication demonstrated favorable circulatory system outcomes [65]. Therefore, adjuvant CHP therapy may be a choice for patients who are unsuitable for oral anticoagulation with antiplatelet therapy.

Our study has several limitations. First, the non-TCM users may have received other complementary therapies during the study. The TCM data from our NHI database is confined to CHPs. Herbal decoctions were not recorded in the database. Second, the TCM users may have received acupuncture therapy during the treatment. Several studies show acupuncture has positive effects on heart rate control. The acupuncture point, PC-6 (Pericardium-6; “Neiguan” in modern Chinese language), is involved in controlling arrhythmias [66–69]. Third, lifestyle-related information such as data on drinking, weight, or smoking status in this cohort was lacking. Fourth, as we discussed previously, there were still some risks of bleeding and adverse effects when we combined CHPs with warfarin, which promotes blood circulation and removes blood stasis. The data for the risk of bleeding and adverse effects in this cohort was lacking. Fifth, the coagulation function and percentage of AF ablation between TCM and non-TCM users were not available in LHID. It was unlikely that we could provide the data on AF burden.

## Conclusions

In this cohort study, TCM users had a lower risk of new onset ischemic stroke and lower hospitalization within 1 year, compared to non-TCM users. Therefore, adjuvant CHPs therapy may be used in AF patients to prevent ischemic stroke.

## Supporting Information

S1 Table Distribution for demographic characteristic between TCM and non-TCM cohort after propensity score matching. (DOCX)

S2 Table Incidence and hazard ratio for ischemic stroke in TCM cohort compared with non-TCM cohort after propensity score matching. (DOCX)

S3 Table Odds ratio for ER and hospitalization due to AF within one year after index date in TCM cohort compared with non-TCM cohort in logistic regression. (DOCX)

S4 Table The Incidence and Hazard Ratios for Ischemic Stroke among the Different Treatments. (DOCX)
