# Supplementary material for: Adjuvant Chinese Herbal Products for Preventing Ischemic Stroke in Patients with Atrial Fibrillation
Source: PLoS One. 2016 Jul 18;11(7):e0159333. doi: 10.1371/journal.pone.0159333 (PMC4948896; doi:10.1371/journal.pone.0159333)
Supplement: S1 Table — (DOCX) [file pone.0159333.s001.docx]

**S1 Table. Distribution for demographic characteristic between TCM and non-TCM cohort after propensity score matching**

|  | Study 1 | | | |  | Study 2 | | | |  |
| --- | --- | --- | --- | --- | --- | --- | --- | --- | --- | --- |
|  | TCM  N=258 | | Non-TCM  N=258 | | p-value | TCM  N=311 | | Non-TCM  N=311 | | *p*-value |
| Gender | n | % | n | % | 0.20 | n | % | n | % | <0.0001 |
| Women | 183 | 70.9 | 196 | 76.0 |  | 236 | 75.9 | 100 | 32.2 |  |
| Men | 75 | 29.1 | 62 | 24.0 |  | 75 | 24.1 | 211 | 67.9 |  |
| Age, year |  |  |  |  | 0.47 |  |  |  |  | <0.0001 |
| 20-44 | 83 | 32.2 | 86 | 33.3 |  | 125 | 40.2 | 66 | 21.2 |  |
| 45-64 | 133 | 51.6 | 121 | 46.9 |  | 144 | 46.3 | 166 | 53.4 |  |
| 65+ | 42 | 16.3 | 51 | 19.8 |  | 42 | 13.5 | 79 | 25.4 |  |
| Mean (SD) | 52.2 | (13.8) | 52.0 | (14.2) | 0.086 | 49.4 | (14.3) | 50.1 | (13.7) | <0.0001 |
| Comorbidity |  |  |  |  |  |  |  |  |  |  |
| Hypertension | 85 | 33.0 | 95 | 36.8 | 0.36 | 86 | 27.7 | 126 | 40.5 | 0.0007 |
| Diabetes | 19 | 7.36 | 26 | 10.1 | 0.27 | 20 | 6.43 | 27 | 8.68 | 0.29 |
| Hyperlipidemia | 77 | 29.8 | 85 | 33.0 | 0.45 | 86 | 27.7 | 82 | 26.4 | 0.72 |
| Ischemic heart disease | 55 | 21.3 | 66 | 25.5 | 0.25 | 56 | 18.0 | 76 | 24.4 | 0.0498 |
| CHF | 6 | 2.33 | 11 | 4.26 | 0.22 | 6 | 1.93 | 28 | 9.00 | 0.0001 |
| Mean CHA2DS2-VASc score (SD) | 1.57 | (1.19) | 1.79 | (1.32) | 0.054 | 1.49 | (1.10) | 1.49 | (1.10) | 1.00 |
| Mean propensity score, (SD) | 0.34 | (0.20) | 0.33 | (0.20) | 0.89 | 0.23 | (0.10) | 0.23 | (0.10) | 1.00 |

Chi-square and t-test

Study 1, matched with age, gender and comorbidity

Study 2, matched with CHA2DS2-VASc score

In study 1, we selected comparisons based on propensity score which counted according to age, gender, and comorbidity (included hypertension, diabetes, hyperlipidemia, ischemic heart disease, and CHF) using logistic regression. In study 2, we selected comparisons based on propensity score which counted according to age, gender, and CHA2DS2-VASc score using logistic regression. The result revealed that TCM users had a lower ischemic stroke risk than non-users in study 1 and study 2, but it did not achieve a significantly different in study 1. The other results in study 1 and study 2 also showed that TCM users had lower risk of hospitalization than non-users.
