# Supplementary material for: Adjuvant Chinese Herbal Products for Preventing Ischemic Stroke in Patients with Atrial Fibrillation
Source: PLoS One. 2016 Jul 18;11(7):e0159333. doi: 10.1371/journal.pone.0159333 (PMC4948896; doi:10.1371/journal.pone.0159333)
Supplement: S2 Table — (DOCX) [file pone.0159333.s002.docx]

**S2 Table. Incidence and hazard ratio for ischemic stroke in TCM cohort compared with non-TCM cohort after propensity score matching**

|  | TCM | | | Non-TCM | | | TCM vs non-TCM HR (95% CI) | |
| --- | --- | --- | --- | --- | --- | --- | --- | --- |
|  | Event no | PY | Rate | Event no | PY | Rate | Mode 1 | Model 2 |
| Study 1 |  |  |  |  |  |  |  |  |
| Overall | 6 | 978 | 6.13 | 23 | 1610 | 14.29 | 0.32 (0.12-0.81)* | 0.44 (0.14-1.44) |
| Gender |  |  |  |  |  |  |  |  |
| Women | 2 | 660 | 3.03 | 19 | 1251 | 15.18 | 0.18 (0.04-0.80)* |  |
| Men | 4 | 318 | 12.58 | 4 | 358 | 11.16 | 0.52 (0.12-2.36) |  |
| Age, year |  |  |  |  |  |  |  |  |
| < 65 | 1 | 801 | 1.25 | 13 | 1356 | 9.58 | 0.13 (0.02-0.99)* |  |
| 65+ | 5 | 177 | 28.27 | 10 | 253 | 39.51 | 0.65 (0.20-2.15) |  |
| Study 2 |  |  |  |  |  |  |  |  |
| Overall | 6 | 1142 | 5.25 | 26 | 1891 | 13.75 | 0.36 (0.15-0.88)* | 0.33 (0.12-0.92)* |
| Gender |  |  |  |  |  |  |  |  |
| Women | 2 | 825 | 2.43 | 8 | 647 | 12.36 | 0.19 (0.04-0.92)* |  |
| Men | 4 | 318 | 12.58 | 18 | 1244 | 14.47 | 0.90 (0.30-2.67) |  |
| Age, year |  |  |  |  |  |  |  |  |
| < 65 | 1 | 966 | 1.04 | 16 | 1502 | 10.65 | 0.10 (0.01-0.80)* |  |
| 65+ | 5 | 177 | 28.27 | 10 | 389 | 25.68 | 1.00 (0.34-2.97) |  |

Model 1, adjusted for age, gender and comorbidity in study 1, and adjusted for CHA2DS2-VASc score in study 2.

Model 2, stratified on matched pair

* *p* < 0.05
