# Supplementary material for: Adjuvant Chinese Herbal Products for Preventing Ischemic Stroke in Patients with Atrial Fibrillation
Source: PLoS One. 2016 Jul 18;11(7):e0159333. doi: 10.1371/journal.pone.0159333 (PMC4948896; doi:10.1371/journal.pone.0159333)
Supplement: S3 Table — (DOCX) [file pone.0159333.s003.docx]

**S3 Table. Odds ratio for ER and hospitalization due to AF within one year after index date in TCM cohort compared with non-TCM cohort in logistic regression**

|  | TCM | | Non-TCM | | TCM vs non-TCM OR (95% CI) | |
| --- | --- | --- | --- | --- | --- | --- |
|  | n | % | n | % | Mode 1 | Model 2 |
| Study 1 |  |  |  |  |  |  |
| ER | 32 | 12.4 | 34 | 13.2 | 0.96 (0.57-1.61) | 0.94 (0.56-1.55) |
| Hospitalization | 2 | 0.78 | 63 | 24.42 | 0.02 (0.01-0.10)*** | 0.02 (0.002-0.12)*** |
| Study 2 |  |  |  |  |  |  |
| ER | 45 | 14.5 | 36 | 11.6 | 1.30 (0.81-2.08) | 1.33 (0.81-2.20) |
| Hospitalization | 2 | 0.64 | 88 | 28.3 | 0.02 (0.004-0.07)*** | 0.01 (0.02-0.08)*** |

Model 1, normal logistic regression adjusted for age, gender and comorbidity

Model 2, conditional logistic regression
