# Supplementary material for: Adjuvant Chinese Herbal Products for Preventing Ischemic Stroke in Patients with Atrial Fibrillation
Source: PLoS One. 2016 Jul 18;11(7):e0159333. doi: 10.1371/journal.pone.0159333 (PMC4948896; doi:10.1371/journal.pone.0159333)
Supplement: S4 Table — (DOCX) [file pone.0159333.s004.docx]

**S4 Table. The Incidence and Hazard Ratios for Ischemic Stroke among the Different Treatments.**

|  |  | | | | TCM vs. non-TCM HR (95% CI) | | |
| --- | --- | --- | --- | --- | --- | --- | --- |
|  | N | Event (no.) | PY | Rate^＃^ | Crude | Model 1 | Model 2 |
| TCM | 311 | 6 | 1143 | 5.25 | 1.00 | 1.00 | 1.00 |
| Non-TCM |  |  |  |  |  |  |  |
| Only warfarin | 424 | 47 | 1888 | 24.89 | 4.84 (2.07-11.3)*** | 2.99 (1.25-7.12)* | 3.36 (1.42-7.92)** |
| Only dipyridamole | 599 | 71 | 3661 | 19.39 | 3.86 (1.67-8.90)** | 2.19 (0.94-5.13) | 2.62 (1.13-6.10)* |
| Only aspirin | 582 | 77 | 3176 | 24.25 | 4.73 (2.06-10.9)*** | 2.54 (1.08-5.95)* | 3.05 (1.31-7.10)** |
| Warfarin + dipyridamole | 21 | 4 | 96 | 41.50 | 8.09 (2.28-28.7)** | 4.06 (1.12-14.7)* | 4.85 (1.35-17.4)* |
| Warfarin + aspirin | 34 | 5 | 158 | 31.55 | 5.90 (1.80-19.3)*** | 3.21 (0.97-10.7) | 4.02 (1.22-13.3)* |
| Dipyridamole + aspirin | 50 | 10 | 333 | 30.07 | 6.00 (2.17-16.5)*** | 3.67 (1.31-10.3)* | 3.99 (1.43-11.1)** |
| All | 5 | 2 | 20 | 97.62 | 19.4 (3.92-96.4)*** | 8.48 (1.64-43.8)* | 11.3 (2.25-53.3)** |

CHA2DS2-VASc, congestive heart failure, hypertension, age ≥75, diabetes mellitus, prior stroke or transient ischemic attack–vascular disease, age 65 to 74, female; HR, hazard ratio; PY, person-year; TCM, traditional Chinese medicine.

Model 1, adjusted for age, sex, and comorbidities.

Model 2, adjusted for the CHA2DS2-VASc score.

* *P*<0.05, ** *P*<0.01, and *** *P*<0.0001.

^＃^ per 1000 person-years

To assess the incidence of ischemic stroke between different treatments, we compared one, two, and three medications. Compared to TCM users, the AF patients who received warfarin, dipyridamole, or aspirin had higher ischemic stroke risk [Table S4; adjusted for age, sex, and comorbidities, HR=2.19 (95%CI=0.94-5.13) to 8.48 (95%CI=1.64-43.8); adjusted for CHA_2_DS_2_-VASc score, HR=2.62 (95%CI=1.13-6.19) to 11.3 (95%CI=2.25-53.3)]. The proportion of AF-related ER visits within 1 year after the AF diagnosis was lower in TCM users than in non-users (14.5% vs. 15.3%, respectively; odds ratio, 0.94 in model 1 and 0.87 in model 2).
